# Life span policies and macroeconomic transition will help the 21st‐century brain health revolution in developing countries

**DOI:** 10.1002/alz.70006

**Published:** 2025-02-24

**Authors:** Cyprian M. Mostert, Chinedu Udeh‐Momoh, Manasi Kumar, Murad Khan, Shehzad Ali, Kendi Muchungi, Gloria Chemutai, Cynthia Smith, Dominic Trepel, Harris Eyre, Lukoye Atwoli, Zul Merali

**Affiliations:** ^1^ Brain and Mind Institute Aga Khan University Nairobi Kenya; ^2^ Department of Population Health Aga Khan University Nairobi Kenya; ^3^ Global Brain Health Institute The University of Dublin Trinity College Institute of Neuroscience Dublin Ireland; ^4^ School of Medicine Wake Forest University Winston‐Salem North Carolina USA; ^5^ Institute for Excellence in Health Equity New York University School of Medicine New York USA; ^6^ Department of Epidemiology and Biostatistics Schulich School of Medicine and Dentistry Western University Ontario Canada; ^7^ Baker Institute's Neuro‐Policy Program Center for Health and Biosciences. Baker Hall MS‐40 Rice University Houston Texas USA

**Keywords:** brain health, developing countries, global brain health framework, lifespan policies, macroeconomic transitions, World Health Organization

## Abstract

In 2022, the World Health Organization (WHO) issued the Intersectoral Global Action Plan for Epilepsy and Other Neurological Disorders for 2022 to 2031, emphasizing important connections between brain health, population well‐being, and economic growth. A year later, the WHO followed up with strategic guidelines aimed at enhancing brain health outcomes in developing countries. However, critical gaps remain. Our policy forum paper advocates for policies that target brain health across all stages of life, starting with measures to reduce the consumption of alcohol, sugar, and tobacco. Additionally, we propose the integration of school meal programs and social pension schemes as essential lifespan policies to safeguard brain health. To support these policies, developing countries must implement key macroeconomic reforms. These include revising international trade agreements, strengthening tax systems, curbing illicit financial flows, eliminating financial exclusions, and expanding social welfare systems. Such reforms are critical for creating an environment that supports long‐term brain health initiatives.

**Highlights:**

- The are critical gaps in the WHO policy framework for brain health.
- We advocate policies that target brain health across all stages of life, starting with measures to reduce alcohol, sugar, and tobacco consumption.
- Additionally, we propose integrating school meal programs and social pension schemes as essential lifespan policies to safeguard brain health.
- To support these policies, developing countries must implement key macroeconomic reforms.
- By adopting these measures, developing countries can lead the charge in advancing the 21st‐century brain health agenda, fostering both societal well‐being and sustainable economic development.

## 1 INTRODUCTION

**FIGURE 1:**
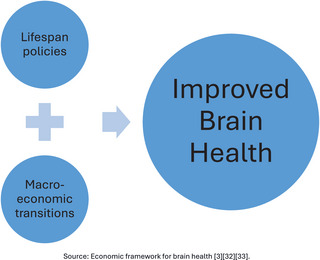
Economic drivers of brain health in developing countries. Source: Economic framework for brain health.^3^, ^32^, ^33^

In 2022, the World Health Organization (WHO) published a discussion paper proposing strategies to enhance brain health from 2022 to 2031.^1^ In 2023, the organization followed this up with strategic guidelines aimed at improving brain health outcomes in developing countries.^2^ However, both documents fell short in addressing key gaps – specifically, the absence of comprehensive, evidence‐based policies considering brain health across the entire lifespan, that is, from perinatal stages and infancy to older ages. This is critical as brain health is shaped by factors at every stage of life, including in utero. Moreover, the guidelines overlooked the need for macroeconomic shifts to support the implementation of local‐level policies, a significant omission given the profound impact of modern economic environments on brain health.^3^ For example, policies aimed at early childhood education, nutrition, and access to healthcare can profoundly shape brain and cognitive development, yet they are often underfunded. Similarly, economic investments in age‐friendly infrastructures and progressive pension systems are essential for promoting cognitive health in older populations, but these services remain largely neglected and unavailable for the most part in developing countries. This policy perspective underscores the urgent need for a life‐course approach to brain health, where policies are designed to support cognitive development and resilience at every age. The paper further highlights the importance of aligning these policies with macroeconomic changes, such as revising international trade agreements, strengthening tax systems, curbing illicit financial flows, eliminating financial exclusions, and expanding social welfare systems. These macroeconomic reforms are necessary to ensure sustainable improvements in brain health for developing countries (Figure 1).

### 1.1 Landscape of brain health in developing countries

The *Lancet*’s 2024 publication reveals that brain health conditions can be categorized into two groups.^4^ The first group includes neurodevelopmental disorders, which are strongly linked to inadequate childhood nutrition. The second group involves late‐life neurodegenerative conditions, which are closely associated with factors like tobacco and alcohol use, poor sleep, depression, and poor prenatal nutrition.^5^ High sugar intake and viral infections significantly worsen brain health conditions such as dementia and stroke.^6^, ^7^ However, existing models for brain health, including those mentioned in the aforementioned reports,^1^, ^2^ fail to fully capture the unique contextual factors at play in developing countries. Some of the recommendations to avert brain ill‐health are not applicable to developing countries.

Brain health conditions are heavily influenced by macroeconomic stressors such as poverty^8^ and inadequate/weak social welfare systems,^9^ all of which exacerbate the burden on brain health. Addressing brain health in these regions requires not only medical interventions but also significant macroeconomic transitions and policy reforms tailored to local contexts.

Currently developing countries face a severe shortage of neurological professionals, with 70 times fewer professionals per 100,000 individuals compared to high‐income countries.^10^ As a result, these regions bear the brunt of the global brain health disease burden, with over 80% of neurological deaths and disability occurring in these regions due to lack of care,^11^ doctors, and relevant specialists. Such scarcity also highlights the urgent need for models that address not only biomedical risk factors but also broader macroeconomic reforms that are unique to developing countries’ brain health initiatives.

### 1.2 Lifespan policies for brain health: contextual approaches for developing countries

National health promotion policies (NHPPs) targeting alcohol, sugar, and tobacco consumption have been identified as powerful tools for improving brain health outcomes.^12^ Additionally, nutrition‐related policies, such as school meal programs, are crucial for fostering cognitive development in children. Evidence of successful implementation of such programs has been reported in developing countries such as Pakistan^13^ and South Africa,^14^ and from global studies.^15^ Similarly, scholars from China,^16^ South Africa,^17^ and Mexico^18^ strongly advocate policies supporting healthy aging, with social pension programs playing a pivotal role in promoting brain health.

In most of these studies, poverty, poor nutrition, and excessive consumption of alcohol, sugar, and/or tobacco are widely recognized as significant drivers of poor brain health outcomes, leading to underdevelopment of cognitive resilience and poor brain health throughout the lifespan.^19^ As a result, NHPPs are seen as cost‐effective interventions that can greatly improve health‐related behaviors and overall brain health outcomes, offering hope for mitigating the negative impacts of underdevelopment, risky behaviors, and cognitive decline. These policies can be financed by aggressive taxation of alcohol, tobacco, and sugar‐sweetened beverages.

Unfortunately, many developing countries are lagging in implementing NHPPs. Some authors argue that governments are unlikely to prioritize these programs,^20^ especially since even global health institutions like the WHO have not provided a comprehensive policy framework on brain health.^1^, ^2^

**FIGURE 2:**
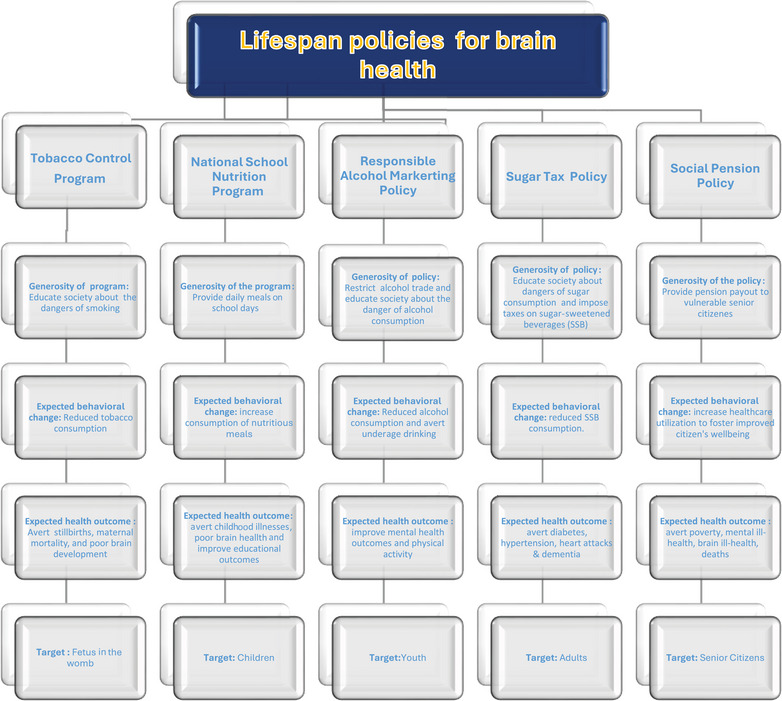
Policy drivers of brain health. Source: Policy framework for health promotion in South Africa.^14^, ^17^, ^21^, ^24^, ^25^, ^27^

So far, developing countries such as South Africa have taken assertive steps by implementing a range of NHPPs, including the Tobacco Control Programme (TCP), National School Nutrition Programme (NSNP) or School Feeding Policy (SFP), Responsible Alcohol Marketing Policy (RAM) (also known as Alcohol Restrictive Policy), Health Promotion Levy Tax (also known as Sugar Tax Policy), and Social Pension Policy (SPP), all adopting a lifespan approach. These policies aim to prevent risky behaviors and promote brain health from early life to old age (Figure 2). For other developing nations, adopting such policies to boost brain and cognitive resilience is essential, especially in contexts where populations face insurmountable and unavoidable stress exposures from factors like wars/conflicts, severe pollution and climate shocks, and economic hardships. Implementing these lifespan policies in a cost‐efficient manner could ensure that the goal of advancing global brain health within the 21st century is not only within reach but an imperative for the social and economic health of nations.

### 1.3 Lessons from South Africa's lifespan policy framework for brain health

#### 1.3.1 Tobacco control program and brain health: policy targeting the fetus

**FIGURE 3:**
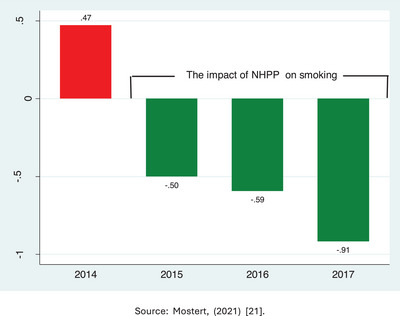
Changes in smoking in low‐income households after implementation of NHPP focusing on controlling tobacco consumption. Source: Mostert, (2021) publication.^21^

In 2015, the South African government implemented the TCP under the national health promotion policy to reduce tobacco consumption as a significant risk factor for fostering brain health conditions.^21^ In 2021, a study was conducted to assess the impact of the policy on tobacco consumption.^21^ The study found that the tobacco control program consistently led to decreases in smoking in low‐income households, as depicted in Figure 3. Additionally, the study revealed that TCP reduced stillbirths by 8.36% in urban areas and 2.84% in rural areas of South Africa. Stillbirths are deaths linked in most cases to brain injury in fetuses.^22^ When looking at foreign nationals residing in South Africa, the study indicated that TCP decreased stillbirths by 7.61% in urban areas and by 2.79% in rural regions.^21^ These comprehensive findings suggest that TCP would greatly benefit developing countries' fetal brain health outcomes.

#### 1.3.2 National school nutrition program and brain health: policy targeting children

School meal programs are widely recognized as a powerful tool to combat hunger and improve the well‐being of underprivileged children. Inadequate nutrition has been linked to stunted brain development,^23^ leading to lower academic performance. A 2021 study in South Africa evaluated the impact of school meal programs on reducing hunger and boosting academic achievement.^14^, ^24^ The results showed significant improvements in academic performance and reduced absenteeism among the students who participated in the program compared to those who did not. Similarly, in Pakistan, school meal programs notably enhanced the cognitive performance of disadvantaged children.^13^ It is crucial for developing countries to prioritize the widespread implementation of school meal programs to promote brain health, especially considering the unequal access to these programs in these regions.

#### 1.3.3 Responsible alcohol marketing policy and brain health: policy targeting youth

**FIGURE 4:**
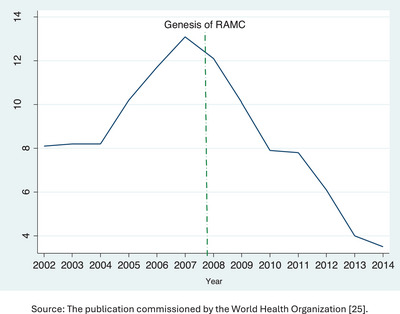
Impact of responsible alcohol marketing campaigns (RAMC) on alcohol advertising in South Africa. Source: The publication commissioned by the World Health Organization.^25^

Recent comprehensive global studies have uncovered strong connections between alcohol consumption and brain health conditions.^19^ This underscores the importance of implementing a responsible alcohol marketing policy to safeguard the well‐being of young brains. In South Africa, the impact of the responsible alcohol marketing policy is evident in the decrease in alcohol advertisements (Figure 4).^25^ Evidence from 46 African countries demonstrates a clear negative correlation between the implementation of alcohol‐restrictive policies and alcohol consumption.^26^ Therefore, it is indisputable that the adoption of this policy is a cost‐effective measure in curbing alcohol consumption, and other developing nations should consider implementing it to prioritize the brain health of young people.

#### 1.3.4 Sugar tax policy and brain health: policy targeting adults

In 2018, South Africa took a bold step by implementing a tax on sugar‐sweetened beverages (SSBs), becoming the first African country to do so, following the lead of Mexico. This proactive policy aims to significantly reduce sugar consumption and mitigate the risk of diabetes, which is a major contributor to brain ill‐health, such as dementia. A 2021 publication provided compelling evidence that the SSB tax has effectively decreased the consumption of SSBs in South Africa.^27^ Notably, between 2016 and 2019, the average volume of taxable SSB purchases plummeted from 518.99 mL per capita per day to 443.39 mL per capita per day.^27^ The far‐reaching impact of this policy is anticipated to substantially affect the prevalence of dementia in South Africa going forward. Recent studies project that the prevalence of dementia among adults aged 65 and older will be 3.8% in 2024 in some regions of South Africa,^28^ down from 11% in 2017,^29^ before the sugar tax policy was not implemented. Indeed, the policy is expected to have positive spillover effects on the brain health of adults.

#### 1.3.5 Social pension policy and brain health: policy targeting senior citizens

The level of income has a significant impact on brain health as well. Studies have shown that individuals with lower incomes have less organized functional brain networks and thinner cortexes compared to those with higher incomes.^30^ In South Africa, reforms that allowed for early pension payouts led to a 3% decrease in depression cases, a 4% decrease in traumatic stress cases, and a 5% decrease in deaths among 60‐year‐old men.^17^ The reforms have also supported healthy cognitive aging. Similar evidence has been observed in other developing countries such as China^16^ and Mexico.^18^ It is crucial for developing countries to consider implementing universal pension systems to support the brain health of senior citizens. Currently, many developing countries have low pension coverage, which undermines efforts to promote the brain health of the elderly population.

### 1.4 Macroeconomic transitions needed to advance brain health

The performance of the macroeconomy plays a significant role in shaping brain health outcomes.^31^ Without substantial transitions in this domain, the lifespan policies discussed earlier will have a limited impact on preventing brain ill‐health. The current literature underscores the need for a global economic shift, from being detrimental to brain health to actively supporting it. Such a transition is critical to accelerating improvements in brain health outcomes,^32^ particularly in developing countries. One of the most urgent transitions needed is the creation of an inclusive global economy that does not perpetuate poverty, underdevelopment, and societal inequities, as these issues are strongly linked to poor brain health outcomes, especially among vulnerable population groups that are often left behind. A global economy that fosters equitable development will lead to healthier populations and improved cognitive health across the lifespan.

**FIGURE 5:**
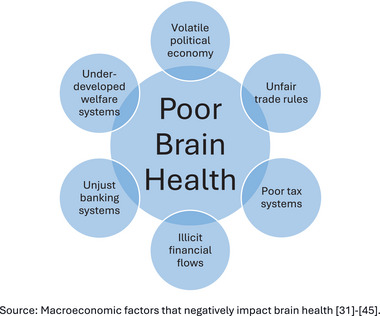
Macroeconomic drivers of poor brain health. Source: Macroeconomic factors that negatively impact brain health.^31^, ^32^, ^33^, ^34^, ^35^, ^36^, ^37^, ^38^, ^39^, ^40^, ^41^, ^42^, ^43^, ^44^, ^45^

We propose five key macroeconomic factors that require immediate transformation in developing countries (Figure 5), as follows:

1. Underdeveloped welfare systems: Strengthening social safety nets is essential for ensuring access to healthcare, nutrition, and education – all of which are crucial for brain health.
2. Inadequate tax systems: Tax reforms are needed to generate sustainable revenue for brain health initiatives and to reduce the wealth gap, which exacerbates disparities in health outcomes.
3. Unfair banking systems: Financial systems must be restructured to provide equitable access to credit and financial services, fostering investments in brain health programs.
4. Trade imbalances between developing countries and the Global North: Addressing these imbalances would allow developing countries to invest in public health infrastructure and brain health programs.
5. Illicit financial flows from developing countries into tax havens: Curbing the illegal transfer of wealth would enable developing nations to retain the much‐needed economic resources required to support positive brain health initiatives.

Such economic transitions will enable developing countries to effectively address poverty, hunger, underdevelopment, and poor brain health outcomes.^33^

In addition to the aforementioned economic factors, a stable political environment is indispensable for advancing brain health. A democratically elected government, functioning parliament, independent judiciary, and efforts to curb corruption are foundational needs. Without political stability, little economic or health progress can be achieved.^34^ For instance, developing countries in Africa, Latin America, South Asia, and the Middle East have struggled to implement long‐term brain health policies due to political unrest and corruption, which inadvertently undermine public health initiatives and prevent sustainable development. Free and fair elections, transparent governance, and the rule of law are vital for creating optimal conditions under which brain health policies can be successfully and sustainably implemented.

#### 1.4.1 International trade rules, poverty, and brain health

Many economic experts have criticized current international trade regulations as institutional fraud designed to impede the economic progress of developing nations.^35^ For instance, when developing countries export to affluent nations in the Global North, they face tariff barriers four times higher than those encountered by wealthy countries exporting to the Global South. These barriers ensure that wealth remains concentrated in rich countries at the expense of developing nations. It is estimated that such trade barriers cost developing countries $100 billion annually. This figure is twice the average aid these countries receive from the Global North.^35^ Undoubtedly, these unjust trade laws perpetuate poverty and hinder maximum economic growth. Poor economic growth results in high unemployment and declining income. These factors are strongly associated with poor mental well‐being, increased rates of common mental disorders, substance‐related disorders, and suicidal behaviors. Hence, reforming international trade laws is crucial for developing countries to achieve economic prosperity and break free from the circle of poor brain health.

#### 1.4.2 Tax system, underdevelopment, and brain health

According to the World Bank, robust tax collection is imperative for economic development to thrive in the 21st century.^36^ The bank argues that governments must collect at least 15% of its gross domestic product as tax to advance developmental goals.^36^ Shockingly, 86% of low‐income and 43% of lower‐middle‐income countries fall short of this threshold.^36^ The situation is even more dire for developing countries affected by fragility, conflict, and violence, as they collected less than 12.6% of GDP as tax in 2023. These countries have experienced high suicide rates and deteriorating mental health conditions. Inadequate tax collection exacerbates poverty and underdevelopment, leading to worsening brain health outcomes. Developing countries urgently need to overhaul their tax systems. Failure to collect taxes undermines the brain health agenda.

#### 1.4.3 Illicit financial flows, poverty, and brain health

Illicit financial flows are illegal transactions that leave developing countries to tax havens of high‐income countries, leading to detrimental social effects, particularly in countries too poor to finance essential public goods such as health.^37^ According to the United Nations, developing countries with high levels of illicit financial flows spend an average of 25% less on health and 58% less on education than countries with low levels of illicit financial flows.^37^, ^38^ Curbing illicit financial flows in Africa could result in an estimated annual gain of $89 billion, which is crucial for financing the underfunded brain health agenda.^38^ If illicit financial flows were effectively addressed in developing countries, approximately 3.9 million women and 190,000 children might receive essential healthcare services.^37^ These estimates highlight the severity of the problem and emphasize the untapped resources in these regions that could be utilized to support brain health initiatives.

#### 1.4.4 Banking system, access to credit, and brain health

Crowdfunded microloans are crucial in financing basic economic activities in developing countries. However, there are concerns about the preferential treatment of women^39^ over men by banks issuing these microloans. This discriminatory banking practice has been linked to triggering domestic violence.^40^

Furthermore, males without access to credit are more likely to die by suicide.^41^ For example, the highest rate of male suicide cases is in Africa,^42^ where discriminatory banking practices are more common. This discriminatory practice is positively associated with the 11 people per 100,000 who die by suicide in Africa. This figure is higher than the global average of nine per 100,000 people.^42^ The male suicide rate in Africa is the highest of all world regions at 18 per 100,000, while the global male average is 12.2 per 100,000 people.^42^ It is evident that the lack of access to credit exacerbates financial worries and psychological distress.^41^ Therefore, it is imperative for developing countries to thoroughly scrutinize discriminatory banking practices in order to advance equitable brain health outcomes.

#### 1.4.5 Welfare system and brain health

The lack of well‐developed social welfare systems in developing countries has a significant negative impact on brain health outcomes.^43^ Recent research shows a strong connection between welfare systems in high‐income countries and the brain health of the population. Countries with social democratic regimes and higher social expenditures tend to have better brain health outcomes compared to countries with lower social expenditures.^44^ Based on this evidence, developing countries urgently need to allocate more resources to social welfare systems to support the brain health agenda of the 21st century. A robust welfare system is crucial, as economic shocks can potentially reverse any progress made in brain health, especially considering that shocks can exacerbate viral conditions,^45^ which can worsen cognitive impairment.

### 1.5 The way forward

**FIGURE 6:**
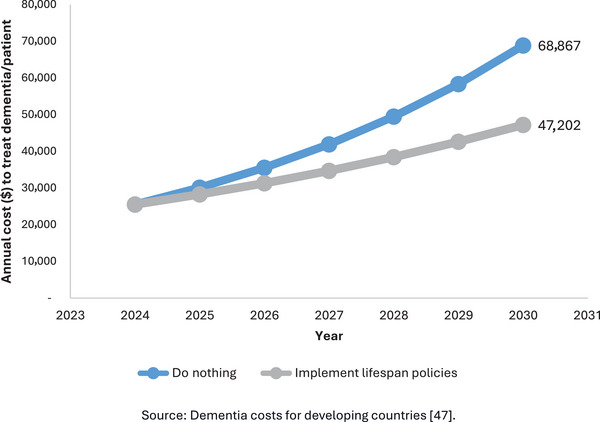
Evolution of dementia costs. Source: Dementia costs for developing countries.^47^

The comprehensive implementation of lifespan policies and macroeconomic transitions recommended in this article is essential if developing countries are to effectively tackle the brain health challenges of the 21st century. Our micro‐costing simulation model, adapted from an Alzheimer's disease progression model,^46^ dementia‐related costs,^47^ and risk factors,^48^ indicates that adopting the proposed preventive lifespan policies could generate significant annual savings of up to $21,000 per patient. Conversely, failure to adopt these strategies could inevitably lead to a substantial rise in dementia‐related hospitalizations and associated costs. Our model projects that the annual cost of treating dementia per patient will increase from $25,510.66 in 2024 to an alarming $68,867.41 by 2030 (Figure 6). These escalating costs underscore the urgent need for intervention. Moreover, evidence suggests that up to 45% of dementia cases can be prevented through the adoption of targeted, progressive, preventive policies.^48^ Policymakers must recognize the substantial long‐term benefits of these strategies, as they have the potential not only to reduce healthcare costs but also to improve the quality of life for millions of people either at risk for or living with a diagnosis of dementia. Achieving the brain health goals of the 21st century is within reach; however, it requires immediate action and commitment to both policy reform and economic transitions.

## 2 CONCLUSION

The currently available global frameworks for promoting brain health^1^, ^2^, ^48^ have notable limitations. For example, the 2023 WHO report highlighted the low level of certainty regarding the effectiveness of psychosocial and non‐pharmaceutical interventions in preventing brain‐related conditions.^2^ Our perspective draws on global evidence to present additional policies for improving brain health that were not sufficiently addressed in the 2023 WHO report.

Furthermore, the heavy emphasis on physical activity improvements as a primary prevention strategy for brain ill‐health, as suggested in the 2023 WHO report,^2^ may not be as effective in developing countries, where the level of physical activity is already high,^49^, ^50^ which may limit its impact on preventing or reducing the impact of brain‐related illnesses. Therefore, focusing solely on physical activity risks – overlooking other critical modifiable risk factors that contribute to poor brain health outcomes – may not advance the 21st‐century brain health revolution.

To truly improve brain health and cognitive resilience in developing countries, governments must take proactive steps to address broader contextual risk factors. These include poverty, income inequality, hunger, underdevelopment, and harmful lifestyle choices such as tobacco, alcohol, and sugar consumption. These factors play a significant role in shaping brain health and resilience, which are essential components of brain capital – a nation's cognitive and emotional resources that drive innovation and economic growth.

If global frameworks and consensus recommendations for reducing the risk of cognitive impairment and dementia are not updated to reflect these broader concerns, there is substantial risk that developing countries will struggle to comprehensively improve brain health outcomes. The current global dementia risk factors for 2024^48^ are biased toward high‐income countries and do not reflect the perspective of developing countries.

Policymakers must look beyond existing frameworks, such as the current WHO guidelines,^1^, ^2^ and adopt a more holistic, life‐course approach. This approach should further incorporate the suggested macroeconomic transitions needed to support brain health. These endeavors are critical for advancing the 21st‐century brain health agenda and ensuring long‐term brain and cognitive resilience, as well as economic sustainability in developing nations.

## CONFLICT OF INTEREST STATEMENT

All authors have nothing to disclose, except Harris Eyre and Dominic Trepel. Harris Eyre has received consulting fees from Kooth LLC, Roche, and Novo Nordisk. Harris Eyre also received payment from the Euro‐Mediterranean Economists Association for teaching and travel support from Harry Z. Yan, Weiman Gao, Robert S Kaplan, Benjamin Cheng, and Winnie Cheng. In addition, Harris Eyre owns stock in Almond Health. Dominic Trepel holds grants from the Health Research Board, Ireland, the Irish Research Council, the National Health and Medical Research Council (Australia), the Global Brain Health Institute, and the Public Health Agency Northern Ireland. Author disclosures are available in the Supporting Information.

## CONSENT STATEMENT

Consent was not necessary for this study.

## Supporting information



Supporting Information
